# Autofluorescent Cancer Stem Cells: Potential Biomarker to Predict Recurrence in Resected Colorectal Tumors

**DOI:** 10.1158/2767-9764.CRC-24-0188

**Published:** 2024-10-02

**Authors:** Sonia Alcala, Gonzalo Serralta San Martin, Marta Muñoz-Fernández de Legaria, Juan Moreno-Rubio, Silvia Salinas, Juan Carlos López-Gil, José Alberto Rojo López, Javier Martínez Alegre, David Abraham Cortes Bandy, Francisco Zambrana, Ana-María Jiménez-Gordo, Enrique Casado, Miriam López-Gómez, Bruno Sainz

**Affiliations:** 1 Department of Biochemistry, School of Medicine, Autónoma University of Madrid and Department of Cancer, Instituto de Investigaciones Biomédicas (IIBm) Sols-Morreale (CSIC-UAM), Madrid, Spain.; 2 Biomarkers and Personalized Approach to Cancer (BIOPAC) Group, Area 3 Cancer, Instituto Ramón y Cajal de Investigación Sanitaria (IRYCIS), Madrid, Spain.; 3 Department of Internal Medicine, Infanta Sofía University Hospital, FIIB HUIS HHEN, Madrid, Spain.; 4 Universidad Europea de Madrid, Madrid, Spain.; 5 Department of Pathology, Infanta Sofía University Hospital, FIIB HUIS HHEN, Madrid, Spain.; 6 Department of Medical Oncology, Infanta Sofía University Hospital, FIIB HUIS HHEN, Madrid, Spain.; 7 Precision Nutrition and Cancer Program, Clinical Oncology Group, IMDEA Food Institute, CEI UAM-CSIC, Madrid, Spain.; 8 Department of General Surgery, Infanta Sofía University Hospital, FIIB HUIS HHEN, Madrid, Spain.; 9 Centro de Investigación Biomédica en Red, Área Cáncer, CIBERONC, ISCIII, Madrid, Spain.

## Abstract

Cancer stem cells (CSC) in colorectal cancer drive intratumoral heterogeneity and distant metastases. Previous research from our group showed that CSCs can be easily detected by autofluorescence (AF). The aim of the present study was to evaluate the potential role of AF CSCs as a prognostic biomarker for colorectal cancer relapse. Seventy-five freshly resected tumors were analyzed by flow cytometry. AF was categorized as high (H-AF) or low, and the results were correlated with histologic features [grade of differentiation, presence of metastases in lymph nodes (LN), perivascular and lymphovascular invasion] and clinical variables (time to relapse and overall survival). Nineteen of the 75 (25.3%) patients experienced relapse (local or distant); of these 19 patients, 13 showed positive LNs and 6 had H-AF. Of note, four of them died before 5 years. Although patients with H-AF CSC percentages in the global population experienced 1.5 times increased relapse [HR, 1.47; 95% confidence interval (0.60–3.63)], patients with H-AF CSC percentages and LN metastases had the highest risk of relapse [HR, 7.92; *P* < 0.004; 95% confidence interval (1.97–31.82)]. These data support AF as an accurate and feasible marker to identify CSCs in resected colorectal cancer. A strong statistical association between H-AF CSCs and the risk of relapse was observed, particularly in patients with positive LNs, suggesting that H-AF patients might benefit from adjuvant chemotherapy regimens and intensive surveillance due to their high propensity to experience disease recurrence.

**Significance:**

AF has been proven to be an accurate biomarker for CSC identification; however, to date, their role as a prognostic factor after resection of colorectal cancer tumors has not been investigated. Our results show that determining the presence of AF CSCs after tumor resection has prognostic value and represents a potentially important tool for the management of patients with colorectal cancer.

## Introduction

Cancer stem cells (CSC), also known as tumor-initiating cells, are responsible for tumor heterogeneity due to their self-renewing capacity and their intrinsic ability to differentiate into different cancer cell lineages (1, 2). Additionally, CSCs are believed to play an important role in the onset of tumor relapse and metastases (3) and are, in large part, the cells responsible for drug resistance and treatment failure (4). Isolation of CSCs is a challenging task, and in the last decades, much effort has been invested in improving our ability to identify this scarce cell population as it is believed that targeting CSCs may improve patient outcome or even eradicate tumors altogether. Although numerous cell surface markers have been validated and used to isolate CSCs from different tumor entities, many require further investigation and no marker has been proven to be a universal CSC biomarker (5). In 2014, we discovered that CSCs of epithelial tumors contain intracellular vesicles coated with the ATP transporter ABCG2, promoting the accumulation of the fluorescent vitamin B2 (i.e., riboflavin) within these vesicles and facilitating the identification of these autofluorescent (AF) cells via fluorescence-based techniques (e.g., flow cytometry; ref. 6). Since then, AF has been used to identify and isolate CSCs across a wide number of cancers (7–10).

The role of CSCs in the development of distant metastases has been the subject of several studies (11); however, to date, no prospective studies have been conducted to resolve this question. Likewise, their precise role as a biomarker for relapse after colorectal cancer surgery remains controversial. In this population of patients, colorectal cancer recurrence usually develops within the first 5 years after primary tumor removal (12), and it is more likely to occur in patients with positive lymph nodes (LN), who may benefit from adjuvant chemotherapy. However, the most appropriate number of chemotherapy cycles required is controversial, and the dichotomy of 3 versus 6 months of therapy is based only on the number of positive LNs and the size of the primary tumor (13). In addition to the presence of positive LNs, postoperative nomograms have been developed to predict the risk of relapse to avoid unnecessary treatment (14); however, these nomograms only include histopathologic parameters and might be inaccurate in some populations such as young patients who usually harbor a more aggressive disease (15).

The incorporation of new and more accurate molecular markers, such as AF, into these nomograms might help in identifying the subgroup(s) of patients with a more aggressive disease and therefore a higher propensity to relapse. Consequently, this patient subgroup could be offered a more intense adjuvant chemotherapy regimen and might benefit from more extensive postsurgery surveillance. The identification of CSCs in resectable colorectal cancer tumors using AF may satisfy this need and could prove to be a very useful tool to individualize the treatment in this population of patients.

The principal objective of this study was to quantify the percentage of AF CSCs in resected tumors of diverse origins; however, as the majority of the tumors analyzed from 2016 to 2022 were colorectal tumors (*n* = 75), we formulated a secondary posteriori objective, which was to evaluate the potential of the percentage of AF CSCs to predict the early recurrence of colorectal cancer. Our secondary endpoints were to establish AF as a feasible technique to identify CSCs from resectable colorectal cancer tumors, to correlate CSC percentages with other clinicopathologic features, and to determine progression-free survival (PFS). To this end, we followed 75 patients with colorectal cancer for 5 years with the aim of determining whether the presence of a high percentage of CSCs correlated with a shorter relapse rate. Indeed, we were able to (i) establish that the presence of a high AF (H-AF) CSC content is related to worse disease progression and (ii) verify that this correlation co-associated with other clinicopathologic or histologic variables, such as positive LNs. The results from our study set the stage for the incorporation of techniques to identify AF CSCs in the clinic to provide important prognostic information after surgery of colorectal tumors.

## Materials and Methods

### Eligible patients

A total of 145 patients with resectable tumors of different entities (Supplementary Table S1) were included in this study. Independent of the tumor type, tumors were numbered from 1 to 145 as they were included in the study. Surgery was performed at the Infanta Sofía University Hospital. All patients signed a written informed consent prior to their inclusion. The study was submitted to the Institutional Review Board (i.e., Ethical Investigation Committee) of La Paz University Hospital, was approved in 2015 (HULP-PI-2112), and was conducted in accordance with the recognized ethical guidelines (i.e., Declaration of Helsinki).

### Data about patients with colorectal cancer

Of the 145 initial patients, 75 patients with resectable colorectal cancer were further studied. Patients with colorectal cancer had a median age of 71 years and an acceptable sex/gender distribution, with 63% being male and 37% being female. Patients were not randomized. By definition, all patients were M0 (they have no metastases), and therefore, there were no stage IV patients. The first patient with colorectal cancer was included in January 2016, and the last patient underwent surgery in 2018. Patients were followed until March 2022 (6 years after the inclusion of the first patient). Demographic and clinicopathologic data were collected at inclusion. Among demographic data, we included gender, age, and date of surgery. Among clinicopathologic data, we included primary localization (right vs. left), tumor–node–metastasis classification (presence of LN + invasion), presence of lymphovascular and perineural invasion, grade of differentiation, presence of microsatellite instability, neutrophil-to-lymphocyte ratio, and levels of hemoglobin, albumin, and creatinine. Patient follow-ups were done every 3 months for the first 2 years, every 6 months from years 2 to 5, and from year 5 onward, every year up to a maximum of 10 years. The presence of locoregional or distant recurrence was examined throughout the first 5 years of follow-up. The primary endpoint of the study was PFS, which was defined as the time from surgical tumor removal until local or distant relapse determined by CT scan.

### Digestion of tumors and detection of CSCs

Following surgery, freshly resected tumors were sent to the Department of Pathology at the Infanta Sofía University Hospital where two expert pathologists analyzed the tumors and selected a representative tumor sample. To avoid compromising the diagnosis of the surgically resected sample and to maintain a consistent tumor volume for all the samples analyzed, a single tumor section of approximately 0.5 to 0.8 cm^3^ for each tumor sample was provided by the Department of Pathology of the Infanta Sofía University Hospital for the analyses detailed below. Samples were transferred to 15-mL conical tubes containing RPMI medium supplemented with 10% FBS (Invitrogen, Cat no. 10270106), 100 units/mL penicillin/streptomycin (Invitrogen, Cat no. 15140122), and 2× fungizone (Invitrogen, Cat no. 15290018); stored at 4°C; and transferred, within 5 hours after surgery, to the Department of Biochemistry at the Autónoma University of Madrid for flow cytometry analysis using a 4-laser Attune NxT Acoustic cytometer (Thermo Fisher Scientific, RRID: SCR_019590). Samples were anonymized, and a blinded analysis was performed. Tumors were mechanically and enzymatically digested with collagenase (STEMCELL Technologies, Cat no. 07902) for 60 minutes at 37°C, clarified via multiple rounds of filter purification with 100 and 40 μm Fisherbrand Sterile cell strainers (Thermo Fisher Scientific, Cat nos. 11517532 and 11587522), centrifuged for 5 minutes at 1,800 rpm, and resuspended as a single-cell suspension in RPMI medium described above. Following an overnight incubation with 100 μmol/L of riboflavin (Sigma) at 37°C, cells were additionally stained with antibodies against the epithelial cell marker/CSC marker EpCAM (anti–hu-EpCAM-APC, mouse monoclonal, dilution: 1:50, Miltenyi Cat no. 130-111-000, RRID: AB_2657497) or the CSC markers CD90 (anti–hu-CD90-APC, mouse monoclonal, dilution: 1:20, Molecular Probes Cat no. A15726, RRID: AB_2534506), CD133 (anti–hu-CD133-APC, mouse monoclonal, dilution: 1:50, Miltenyi Cat no. 130-111-080, RRID: AB_2654889), SSEA1 (anti–hu-SSEA1-APC, recombinant antibody, dilution: 1:50, Miltenyi Cat no. 130-118-671, RRID: AB_2733289), SSEA4 (anti–hu-SSEA4-APC, mouse monoclonal, dilution: 1:50, BioLegend Cat no. 330418, RRID: AB_2616819), or leucine-rich repeat-containing G protein–coupled receptor 5 (LGR5; anti-LGR5, rat, dilution 1:50, BD Pharmingen Cat no. 562731, RRID: AB_2737752), and finally resuspended in flow buffer [1× PBS; 3% FBS (v/v); and 3 mM EDTA (v/v)]. For the detection of LGR5, the secondary goat anti–rat AF546-conjugated antibody (Invitrogen Cat no. A11081, RRID: AB_2534125) was used. Stained cells were analyzed by flow cytometry using an Attune NxT Acoustic cytometer (Thermo Fisher Scientific, RRID: SCR_019590), eliminating aggregates, debris, and dead cells, the latter with 2 µg/mL 4′,6-diamidino-2-phenylindole (DAPI, Sigma). Allophycocyanin (APC) and A546 fluorescence were detected using the filter (Ex637nm/Em660/14) RL1.

**Figure 1:**
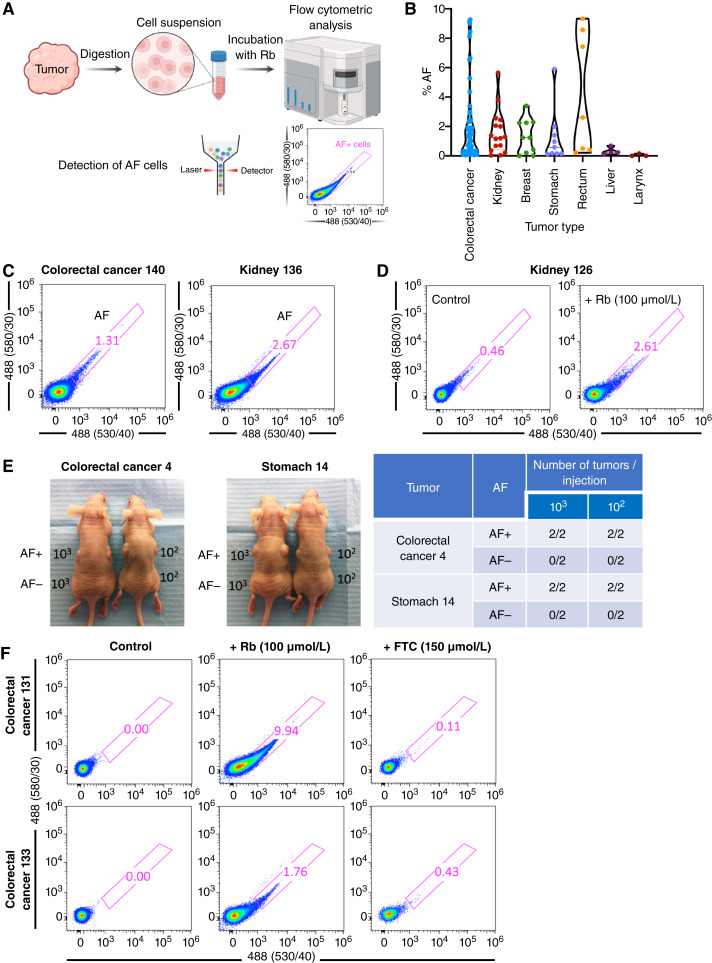
Expression of AF in different tumor entities. **A,** Workflow followed for the detection of AF cells. Tumors were digested and single-cell suspensions incubated overnight with riboflavin (Rb) prior to flow cytometric analysis of AF using a 4-laser Attune NxT Acoustic cytometer. **B,** Distribution of the percentage of AF-positive cells in the indicated tumors (colorectal cancer, *n* = 60; kidney, *n* = 16; breast, *n* = 10; stomach, *n* = 9; rectum, *n* = 7; liver, *n* = 4; larynx, *n* = 3). **C,** Representative flow cytometric plots depicting the percentage of AF cells in colorectal cancer and kidney tumors. **D,** Representative flow cytometric plots depicting the percentage of AF cells in a kidney tumor incubated with and without 100 μmol/L Rb. **E,** Left, Representative images of tumor formation achieved with 10^3^ and 10^2^ AF-positive (+) or AF-negative (−) FACS-sorted cells, injected subcutaneously (in duplicate) in athymic nude mice. Photographs correspond to 12 weeks after injection. Right, Table summarizing the number of tumors formed/number of injections per dilution for a colorectal cancer or stomach tumor, digested and sorted for AF cells. **F,** To confirm that the AF detected was a result of riboflavin accumulation in ABCG2-coated vesicles, parallel samples were incubated overnight (in parallel with Rb) with 150 µM FTC, an ABCG2 inhibitor. AF diminished in FTC-treated cells, confirming that AF is ABCG2 mediated.

For AF detection, cells were excited with blue laser (BL) at 488 nm and selected as the intersection with emission filters 530/40 (BL1) and 580/30 (BL2). Unstained and/or isotype control antibodies were used to set all gates. Approximately 50,000 events (i.e., tumor cells) were analyzed for each tumor. Data were analyzed using FlowJo (RRID: SCR_008520) v9.3 software (Tree Star Inc.). Fumitremorgin C (FTC; Cayman Chemicals, Cat no. 11030), a specific inhibitor of ABCG2, was used overnight (in parallel with Rbf) at a concentration of 150 μmol/L to verify the specificity of the AF observed as described in (6). For a tumor to be considered AF positive, two criteria needed to be met: (i) an AF peak following riboflavin incubation needed to be present and (ii) the AF peak had to disappear or be reduced upon FTC treatment. Please refer to Fig. 1 for representative cytometry plots depicting the AF peak and its disappearance following FTC treatment. Only tumors that met both criteria were considered to contain AF cells.

For cell sorting, a FACSVantage SE flow cytometer was used, and the data were analyzed using BD FACSDiVa software (RRID: SCR_001456).

### Limiting dilution analysis

For assessing tumorigenic potential of the sorted AF− and AF+ tumor cell subsets, dilutions of sorted cells (10^3^ and 10^2^) were injected into the flanks of athymic forkhead box N1; nude mice (Janvier Labs, Cat no. SM-ATH-F, RRID: IMSR_RJ:ATHYMIC-NUDE). Cells were embedded in a mixture 1:1 medium:Matrigel (Corning) in a final volume of 50 µL. Animals were examined twice a week for tumor formation for up to 10 weeks and were sacrificed when the diameter of the tumor reached 1 cm^2^. All *in vivo* procedures in mice were conducted in accordance with protocols approved by the Use Committee for Animal Care from the Universidad Autónoma de Madrid (Ref# CEI-60-1057-A068) and the Comunidad de Madrid (PROEX 335/14). For all *in vivo* experiments, mice were housed according to the institutional guidelines, and all experimental procedures were performed in compliance with the institutional guidelines for the welfare of experimental animals and in accordance with the guidelines for Ethical Conduct in the Care and Use of Animals as stated in The International Guiding Principles for Biomedical Research Involving Animals, developed by the Council for International Organizations of Medical Sciences.

### Immunofluorescence and confocal microscopy

For formalin-fixed, paraffin-embedded blocks, 5-µm sections were cut, and deparaffination of sections and antigen retrieval with 1 mM EDTA were performed following standard protocols. After incubation with blocking solution (PBS 1×, 1% BSA, and 2% FBS), primary antibodies for EpCAM 1:100 (Abcam, Cat no. ab71916, RRID: AB_1603782), CD90-FITC 1:100 (Miltenyi, Cat no. 130-117-684, RRID: AB_2784296), or LGR5 1:100 (BD Pharmingen, Cat no. 562731, RRID: AB_2737752) were added and incubated overnight at 4°C. Subsequently, they were incubated at 37°C for 1 hour with the following secondary antibodies: donkey anti–rabbit AlexaFluor555 1:200 (Invitrogen, Cat no. A31572, RRID: AB_162543), donkey anti–mouse AlexaFluor488 1:200 (Invitrogen, Cat no. A21202, RRID: AB_141607), or goat anti–rat AlexaFluor647 1:200 (Invitrogen, Cat no. A21247, RRID: AB_141778) and DAPI. Samples were mounted in ProLong (Invitrogen, Cat no. P36970), and images were obtained using a Confocal Laser Scanning Microscope LSM710 (Zeiss, RRID: SCR_018063). Images were analyzed in ImageJ software (RRID: SCR_003070).

### Statistical analysis

In this study, continuous variables are described using means and SDs and categorical variables expressed as number and percentage. Box plots of relapse considering AF levels, EpCAM levels, and EpCAM/AF levels were generated. CD90 did not show a normal distribution so it was excluded from the rest of the analyses. Considering that the presence of metastasis in LNs is one of the most powerful markers of recurrence in colorectal tumors, we generated additional box plots of relapse considering the three markers according to the presence or absence of LN invasion. In LN-positive patients, we performed an ROC curve analysis with every CSC marker to obtain the best value to discriminate between patients with relapse and no relapse. We used the Youden index for this purpose. Patients with a value above the average obtained were categorized as high (H-CSC marker) and those with values below average as low (L-CSC marker). The AUC was 0.63 [95% confidence interval (CI), 0.41–0.84] for AF, 0.6 (95% CI, 0.39–0.81) for EpCAM, and 0.58 (95% CI, 0.37–0.79) for EpCAM/AF.

We calculated cumulative incidence and incidence rate of relapse in the global sample and according to LN positivity and CSC marker percentage levels. Kaplan–Meier curves were used to plot survival. We fitted a Cox proportional hazards model including the covariables of interest using a step forward process. The data were analyzed using the statistical package software Stata (RRID: SCR_012763) version 13.0 (StataCorp).

### Data availability

Raw data for this study were generated at the Instituto de Investigaciones Biomédicas and the Infanta Sofía University Hospital. Derived data supporting the findings of this study are available from the corresponding author upon request.

## Results

### The presence of AF across different tumors

AF is the result of riboflavin accumulation in discrete cytoplasmic vesicles overexpressing the ATP-dependent transporter ABCG2 in CSCs (6). The concentration of this fluorescent vitamin therefore generates AF, which can be monitored by live fluorescence imaging or flow cytometry. The ease and utility of such a marker for the identification of CSCs in freshly resected tumors lend itself well to the clinical setting as AF detection is independent of antibodies and can be detected in fresh tissue. As our previously published study characterized AF CSCs primarily in the context of pancreatic ductal adenocarcinoma, with some additional analyses performed in other tumor types such as colon, liver, and lung (6), as a first approximation, we wanted to initially determine in which tumor entities could AF be detected. As such, from 2016 to 2018, 145 surgically-resected tumors at the Infanta Sofía University Hospital were analyzed for the presence of AF cells by flow cytometry (Supplementary Table S1), as described in the “Materials and Methods” section and Fig. 1A. The majority of the tumors analyzed were of gastrointestinal origin, with colorectal cancer being the most representative tumor (*n* = 75 tumors).

Of the 145 tumors analyzed, AF cells were detectable in the majority of tumors (Fig. 1B). Shown in Fig. 1C are representative cytometry plots of AF-positive cells from colon and kidney tumors. Of note, although AF cells could be detected in some tumors (∼5%) in the absence of riboflavin, the levels of riboflavin in blood are lower than those present in the culture medium (1.06–6.38 × 10^−1^ μmol/L in blood vs. 531.40 μmol/L in RPMI medium), and thus, supplementation of digested tumor cell suspensions with riboflavin (100–200 μmol/L) facilitates its concentration in CSCs and subsequently AF detection by flow cytometry (Fig. 1D). Importantly, although we previously showed, using the same protocol, that AF-positive cells isolated from primary colorectal cancer, hepatocellular carcinoma, and non–small cell lung cancer tumors have tumor-initiating capacity (i.e., a CSC-defining characteristic; ref. 6), herein we performed a tumor take study for one colorectal cancer and one stomach tumor, injecting 10^2^ and 10^3^ AF+ and AF− FACS-sorted cells, respectively, into athymic forkhead box N1; nude mice to determine their tumorigenic potential. Although such tumor-initiating studies could not be performed with all of the tumors analyzed in this study, we showed for these two tumors that AF+ cells have increased tumorigenic potential (Fig. 1E). Likewise, the AF detected was ABCG2-mediated, as simultaneous treatment of all tumor digestions with riboflavin and FTC (16) reduced the appearance of AF cells (Fig. 1F), confirming that the AF detected in all tumors is identical to that described previously (6). Taken together, these data support the conclusion that AF cells detected in primary tumors are *bona fide* CSCs.

**Figure 2:**
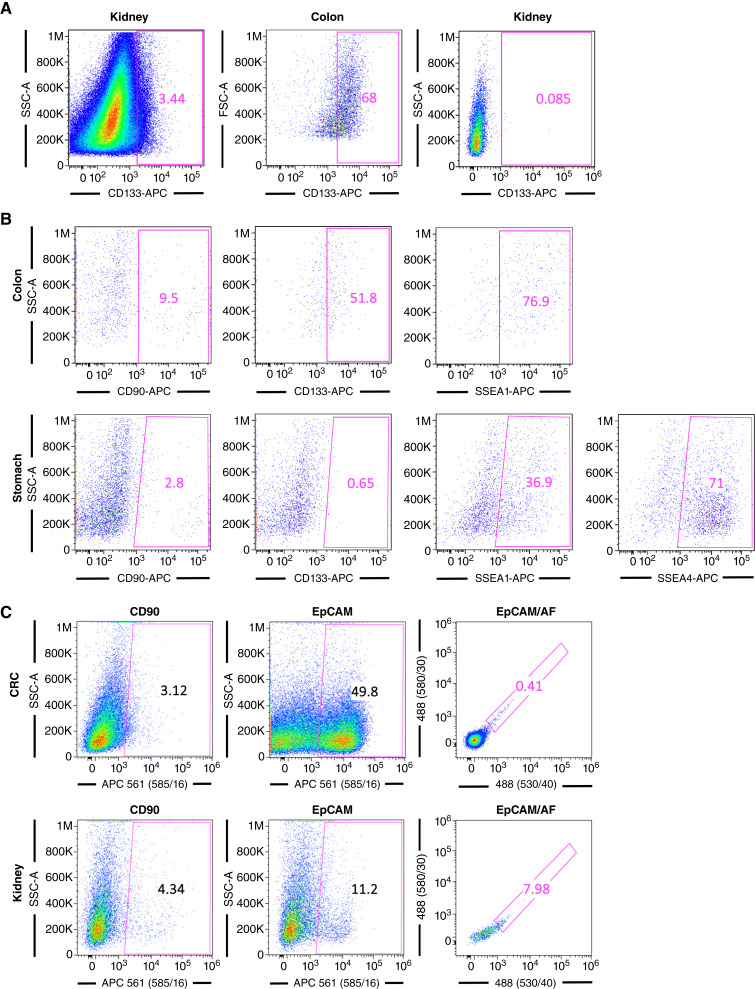
Expression of AF and CSC markers in different tumor entities. **A,** Representative flow cytometric plots depicting the percentage of CD133 cells in one colorectal cancer tumor and two kidney tumors. **B,** Representative flow cytometric plots depicting the percentage of CD90, CD133, SSEA1, and/or SSEA4 cells in colorectal cancer and stomach tumors. **C,** Representative flow cytometric plots depicting the percentage of CD90, EpCAM, and EpCAM/AF cells in colorectal cancer and kidney tumors.

**Figure 3:**
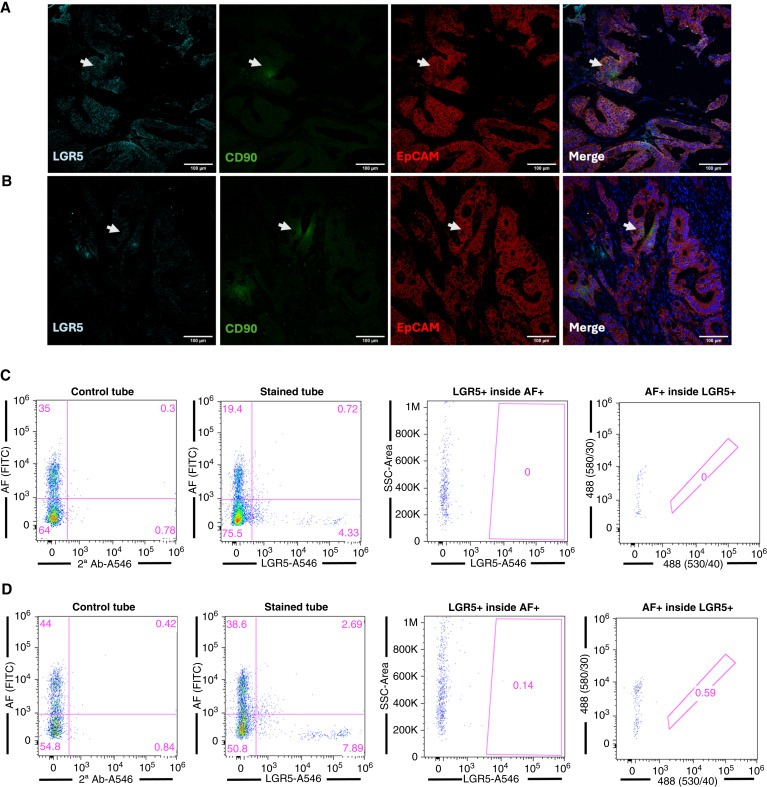
Expression of CSC markers in colorectal cancer. **A** and **B,** Representative confocal images of two colorectal cancer tumors showing CSC marker–expressing cells; cyan, LGR5; green, CD90; red, EpCAM; blue, DAPI (nuclear marker). Arrowheads indicate colocalization. **C** and **D,** Left two plots, representative flow cytometric plots depicting the percentage of AF- and LGR5-positive cells in two colorectal cancer tumors. Control tube, only secondary A546 added. Right two plots, analysis of the percentage of LGR5+ cells inside the AF+ population and vice versa.

In addition to AF, we also included the markers CD90, CD133, SSEA1, and SSEA4 (17, 18), known CSC markers, as well as EpCAM (CD326), which is a marker of epithelial cells and overexpressed in CSCs for many tumor types (19, 20), including colorectal cancer (21–24). Of the traditional CSC markers tested early on across approximately 10 to 20 tumors, CD133 showed inconsistent results, identifying in some cases an apparent small CSC population, in other cases a large (>50%) population of marker-positive cells, or in the majority of cases not identifying a reliable population by flow cytometry (Fig. 2A). Similar inconsistencies were observed for the CSC markers SSEA1 and SSEA4, for which a high expression was observed (Fig. 2B). The only CSC marker that was consistently reliable across all tumor entities was CD90, and thus, the remaining tumors (up to 145) were analyzed for AF and CD90, as well as EpCAM (Fig. 2B and C). In addition, AF was combined with EpCAM to determine the benefit of combining AF with this epithelial cell/CSC marker (Fig. 2C). Of note, after analyzing 121 tumors, we observed a bias toward colorectal tumors. Knowing that colorectal cancer tumors contain LGR5-positive CSCs (25), we analyzed the presence of LGR5+ cells in two colorectal cancer tumors by immunofluorescence (together with CD90 and EpCAM) or by flow cytometry (together with AF; Fig. 3). We validated that LGR5, CD90, and EpCAM could be detected by immunofluorescence confocal microscopy, and CD90 colocalizes with some LGR5+ cells (Fig. 3A and B). Surprisingly, we observed that although LGR5+ cells could be efficiently identified by flow cytometry, they did not overlap with AF+ cells (Fig. 3C and D).

### The presence of CSCs in resectable colorectal tumors

As mentioned above, as the majority of the tumors analyzed in the larger cohort were colorectal cancer, we refocused the study on the 75 localized colorectal cancer tissues obtained after surgery from 2016 to 2018. Patients had a median age of 71 years, and 63% of the patients were male. The rate of positive LNs at the time of surgery was 49%, and 18%, 36%, and 49% of patients were at stage I, stage II, and stage III, respectively. Nineteen patients experienced a relapse (25.3%). Relapsed patients were at stage III and had more positive LN involvement, a higher histologic grade, or the presence of perineural infiltration. Demographic and clinicopathologic data from all patients are shown in Supplementary Table S2.

**Figure 4:**
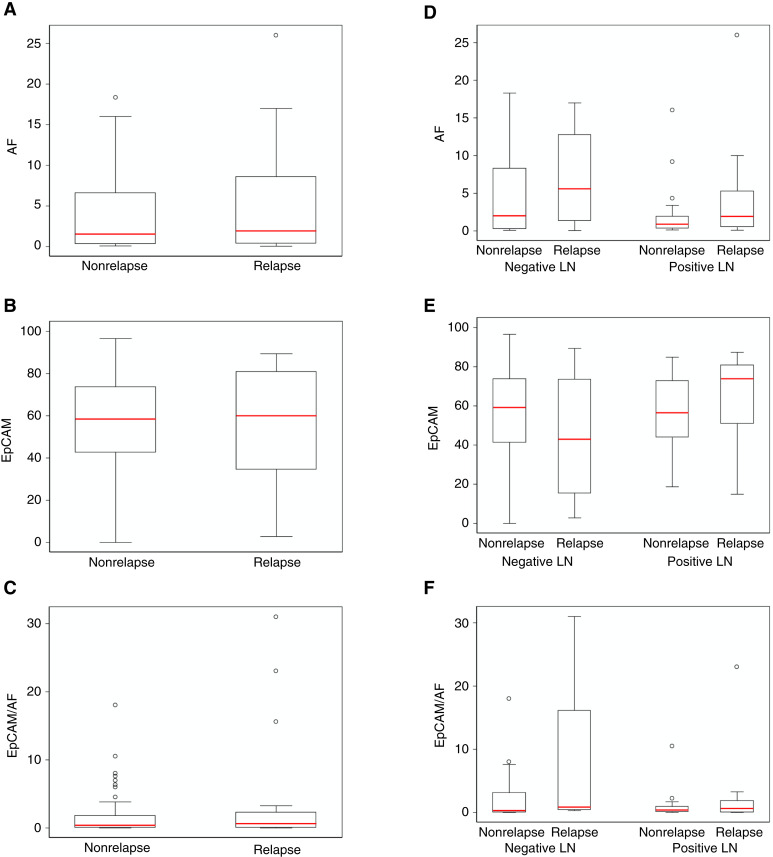
Expression of the CSC markers AF, EpCAM, and EpCAM/AF in patients with colorectal cancer with or without relapse. **A–C,** Box plots according to AF, EpCAM, and EpCAM/AF expression levels in patients with or without relapse. **D–F,** Box plots according to AF, EpCAM, and EpCAM/AF expression levels including LN involvement. No statistically significant differences were found between groups.

The percentage of tumor cells expressing AF, CD90, EpCAM, and EpCAM/AF was determined by flow cytometric analysis as described above. CD90 did not show a normal distribution, so it was excluded from the analysis. AF, EpCAM, and EpCAM/AF percentages by outcome (nonrelapse vs. relapse) were higher in relapsed patients as shown in Supplementary Table S2. In addition, we represented the graph of the CSC percentages as box plots (Fig. 4A–C), and no statistically significant difference between the outcome and the percentage of CSCs, irrespective of the marker used, was observed. As the presence of positive LNs is considered the main prognostic factor of relapse, a subgroup analysis was made according to positive versus negative LNs (Fig. 4D–F). Although CSC marker percentages were higher in the subgroup of patients with positive LNs, there were no statistically significant differences.

### The presence of AF CSCs correlates with cumulative incidence of relapse and incidence rate

We next performed a ROC curve analysis to determine suitable cutoff points for relapse risk stratification according to the expression of each CSC marker or combination. From the ROC curve analysis, we established the following cutoff points: 1.93 for AF percent positive, 73.9 for EpCAM percent positive, and 5.8 for EpCAM/AF percent positive. AF was the CSC marker that demonstrated the best accuracy (71.43%). When comparing H-AF versus low (L)-AF samples, we observed that the H-AF population showed higher histologic grade and pathologic perineural infiltration, even after correcting for the rate of positive LNs. Clinicopathologic data of patients according to high or low percentage levels of AF are shown in Table 1.

**Table 1:** Clinicopathologic characteristics of patients according to AF levels

Patient characteristic	Total sample *n* = 75	Autofluorescence		*P* value
		Low (<1.93) *n* = 45	High (>1.93) *n* = 30	
Sex, *n* (%)				
Female	28 (37)	16 (36)	12 (40)	ns
Male	47 (63)	29 (64)	18 (60)	ns
Age, median (SD)	71 (12)	71 (12)	72 (11)	ns
Tumor type, *n* (%)				
Colon				
Right	34 (47)	20 (47)	14 (48)	ns
Left	38 (54)	23 (55)	15 (52)	ns
Transversal	3 (4)	2 (4)	1 (3)	ns
Tumor stage, *n* (%)				
Stage I	13 (18)	6 (14)	7 (24)	ns
Stage II	27 (36)	14 (31)	13 (43)	ns
Stage III and N1	35 (49)	25 (60)	10 (34)	*P = 0.038*
T factor, *n* (%)				
T1	4 (6)	2 (5)	2 (7)	ns
T2	17 (23)	9 (20)	8 (27)	ns
T3	47 (66)	31 (74)	16 (55)	ns
T4	7 (10)	3 (7)	4 (14)	ns
Tumor histology, *n* (%)				
High histologic grade	5 (7)	2 (5)	3 (10)	ns
Perivascular invasion	11 (15)	5 (12)	6 (21)	ns
Perineural infiltration	3 (4)	2 (5)	1 (4)	ns
Microsatellite instability	5 (8)	3 (8)	2 (7)	ns
CSC levels percent positive (IQR)				
AF	1.62 (0.3–7)	0.41 (0.2–1.4)	8.51 (4–11)	*P < 0.001*
CD 90	2.17 (1–6)	2.4 (1.5–5.5)	1.8 (0.8–5.8)	ns
EpCAM	58.7 (42–74)	58 (41–74)	59 (44–77)	ns
EpCAM/AF	0.48 (0–2)	0.1 (0–0.5)	2.3 (0.6–6.4)	*P* < 0.001

Subsequently, we performed a cumulative incidence of relapse (CIR) and incidence relapse rate analysis (Table 2). The CIR values in the global sample and patients with positive LNs were 25.3% (95% CI, 15.9–36.7) and 37.1% (95% CI, 25.5–55), respectively. Patients with H-CSC marker expression also showed an increased CIR of 30% (95% CI: 14.7–49.4) for H-AF, 36.8% (95% CI: 16.3–61.6) for H-EpCAM, and 30% (95% CI, 11.9–54.3) for H-EpCAM/AF. The CIR in patients with positive LNs and H-CSC levels was 60% (95% CI, 26.2–87.8) for H-AF, 55% (95% CI, 23.4–83.3) for H-EpCAM, and 66.7% (95% CI, 22.3–95.7) for H-EpCAM/AF. The incidence rate of relapse in the global sample was 6.8 per 1,000 patients-month (95% CI, 4.3–10.7), increasing to 38.2 per 1,000 patients-month (95% CI, 17.2–85) in patients with positive LNs and H-AF levels and to 22.5 per 1,000 patients-month (95% CI, 10.1–50) in patients with positive LNs and H-EpCAM levels. Interestingly, in patients with positive LNs and H-EpCAM/AF levels, the incidence of relapse was 68.6 relapses per 1,000 patients-month (95% CI, 25.8–182.9). However, these results were not statistically significant.

**Table 2:** Cumulative incidence and incidence rate for relapse

Parameters	Cumulative incidence			Incidence rate		
	%	95% CI	*P* value	Rate^a^	95% CI	*P* value
Total sample	25.3	15.9–36.7	ns	6.8	4.3–10.7	ns
LN biopsy (N1)						
Positive (+)	37.1	21.5–55	ns	12.3	7.2–21.2	ns
Negative (−)	11.4	3.1–26	ns	2.5	0.9–6.7	ns
AF level						
High	30	14.7–49.4	ns	8	4.1–15.3	ns
Low	22.2	11.2–37.1	ns	6	3.2–11.2	ns
EpCAM level						
High	36.8	16.3–61.6	ns	9.8	4.6–20.6	ns
Low	21.4	11.6–34.4	ns	5.8	3.3–10.2	ns
EpCAM/AF level						
High	30	11.9–54.3	ns	8	3.58–17.75	ns
Low	23.6	13.2–37	ns	6.4	3.7–11	ns
Patients with + LN and % AF						
High	60	26.2–87.8	ns	38.2	17.2–85	ns
Low	28	12–49.4	ns	7.8	3.7–16.4	ns
Patients with + LN and % EpCAM						
High	55	23.4–83.3	ns	22.5	10.1–50	ns
Low	29	12.6–51.1	ns	8.8	4.2–18.7	ns
Patients with + LN and % EpCAM/AF						
High	66.7	22.3–95.7	ns	68.6	25.8–182.9	ns
Low	31	15–50.8	ns	9	4.7–17.4	ns

a Relapses per 1,000 patients-month.

### The presence of AF CSCs is a prognostic biomarker of recurrence in patients with colorectal cancer

**Figure 5:**
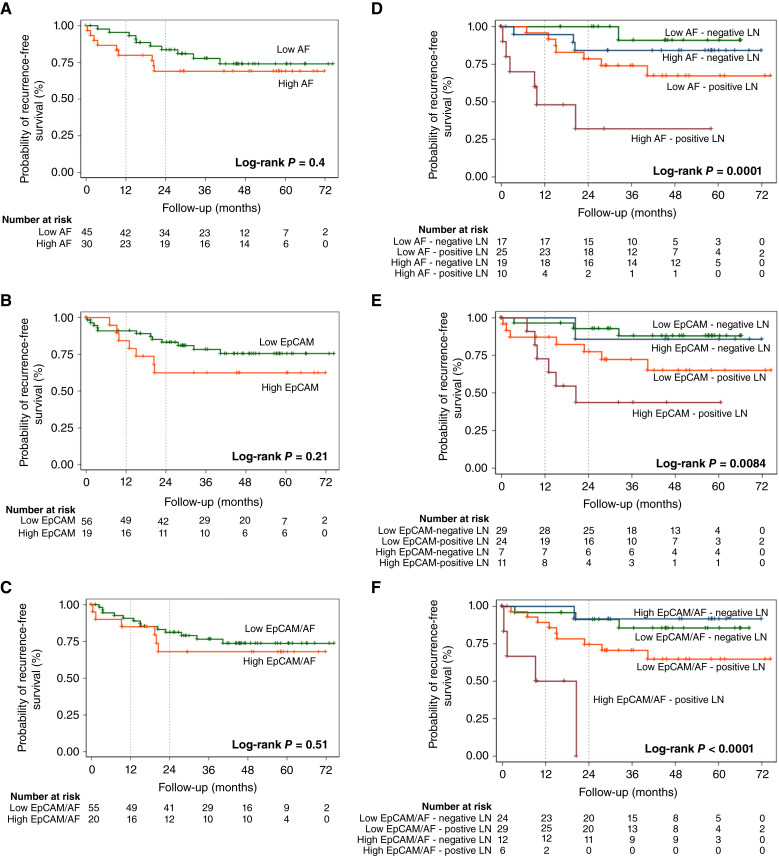
Recurrence-free survival. **A–C,** Kaplan–Meier estimates of recurrence‐free survival in global samples according to AF, EpCAM, and EpCAM/AF expression levels. **D–F,** Kaplan–Meier estimates of recurrence‐free survival in global sample according to AF, EpCAM, and EpCAM/AF expression levels including LN involvement.

Finally, we analyzed relapse-free survival for high versus low percentage levels of each CSC marker in the global sample and in the subgroup of patients with positive LNs (Fig. 5). In the global sample, high versus low percentage levels of each CSC marker did not show any significant difference in recurrence-free survival (Fig. 5A–C); however, patients with positive LNs and H-CSC percentage levels showed significantly worse PFS with H-AF and H-EpCAM/AF outperforming H-EpCAM alone (Fig. 5D–F).

A Cox proportional hazards regression model corroborated these results and demonstrated that patients with H-AF, H-EpCAM, and H-EpCAM/AF were 1.47 (95% CI, 0.6–3.63), 1.8 (95% CI, 0.71–4.57), and 1.38 (95% CI, 0.42–3.63) times more likely to relapse, respectively; however, these ratios were not significant (Table 3). Importantly, when the HR was adjusted for positive LNs and T4 factor, a clear selectivity for AF alone as the better CSC marker was observed, demonstrating that patients with H-AF were 4.06 (95% CI, 1.41–11.64; *P* = 0.009) times more likely to relapse. Patients with H-EpCAM alone or H-EpCAM/AF were 2.47 (95% CI, 0.90–6.80) or 2.72 (95% CI, 0.84–8.84) times more likely to relapse, respectively (Table 3). Model adjustment for sex, age, tumor location, histologic grade, and presence of microsatellite instability was not substantially modified. Taken together, the sum of the results demonstrates that AF represents a strong biomarker that can be used together with LN positivity to better identify patients with an increased probability of disease relapse.

**Table 3:** HR for relapse

Parameters	HR	95% CI	*P* value
Crude HR			
H-AF	1.47	0.60–3.63	ns
H-EpCAM	1.8	0.71–4.57	ns
H-EpCAM/AF	1.38	0.42–3.63	ns
Adjusted HR^a^			
H-AF	4.06	1.41–11.64	*P* = 0.009
H-EpCAM	2.47	0.90–6.80	ns
H-EpCAM/AF	2.72	0.84–8.84	ns

a Adjusted HR for positive LNs and T4 factor. Meeting all assumptions of Cox regression.

## Discussion

The starting hypothesis of this study was that AF CSCs could be detected in different tumor entities. Upon validating that hypothesis, we sought out to determine whether the presence of a higher percentage of AF CSCs in resected colorectal tumors was associated with a lower PFS. We also wanted to elucidate whether a higher percentage of AF CSCs correlated with other clinical or pathologic variables. We demonstrate herein that tumors with H-AF correlate with an increased probability of relapse after colorectal surgery and that this correlation was higher in the subgroup of patients with positive LNs.

The International Agency for Research on Cancer’s GLOBOCAN 2022 data estimates 1,142,286 new colorectal cancer cases and approximately 538,167 deaths (https://gco.iarc.fr/today/home). Surgical resection remains the primary treatment for stage I to III tumors, with prognosis influenced by various pathologic, clinical, molecular, and histologic factors, such as histologic grade, tumor–node–metastasis staging, preoperative carcinoembryonic antigen levels, mismatch repair deficiency status, and presence of CDX2 (26–32). Not even the Oncotype DX Colon Cancer Assay (a 12-gene score that might predict colorectal cancer recurrence) can be universally adopted as it lacks external validation (33). Additional molecular markers like microRNAs and ctDNA show potential: persistent ctDNA postsurgery identifies patients with minimal residual disease at risk of relapse, but this approximation also needs further validation (34). High-risk factors in stage II colon tumors, as defined by American Society of Clinical Oncology, National Comprehensive Cancer Network, and European Society of Medical Oncology, include T4 tumors, insufficiently sampled nodes, poor differentiation, and lymphovascular invasion. Despite these adverse factors, the benefit of chemotherapy for high-risk stage II tumors remains unclear and controversial, emphasizing the need for reliable prognostic markers that can better predict relapse and stratify patients.

In the last decades, much effort has been dedicated to categorizing colorectal tumors in four relevant molecular subtypes with different features (microsatellite instability-like, canonical, metabolic, and mesenchymal; ref. 35). These subtypes are associated with marked differences in survival, supporting the clinical importance of incorporating molecular studies to assess the heterogeneity of colorectal cancer (36), and in metastatic colorectal cancer, these subtypes have become an important tool for physicians to determine the type of systemic chemotherapy or targeted therapy suited for a specific patient (37). However, to date, this molecular classification is of limited help in the adjuvant setting, and physicians rely on the aforementioned clinicopathologic prognostic markers to decide whether adjuvant chemotherapy should be administered in resected tumors. Nonetheless, daily practice demonstrates that a percentage of “potentially good prognosis tumors”—considering some of the above presented parameters—show an early relapse, even though many of these patients were previously precluded from receiving chemotherapy as they did not exhibit the traditional proven factors associated with relapse.

CSCs are considered drivers of tumor growth, metastasis, and chemoresistance and have therefore become one of the main therapeutic targets of interest for cancer-related drug discovery (38, 39). Isolation of CSCs represents one of the major challenges in the field, with the use of surface biomarkers as the main method of detection and isolation (4); however, as CSCs share characteristics with normal stem cells, the challenge is to identify markers specific for CSCs or explore new and more accurate methods for their identification. Riboflavin (also known as vitamin B2) has been suggested to be involved in the progress of colorectal cancer, although the underlying mechanism is unknown (40). Likewise, riboflavin deficiency has been implicated in tumor growth inhibition in animal models (41), and riboflavin supplementation is related to proliferation, invasion, and migration of cancer cells (42). Along these lines, we previously showed that riboflavin accumulates in discrete cytoplasmic vesicles overexpressing the ATP-dependent transporter ABCG2 in epithelial CSCs, allowing for their easy identification via flow cytometric detection and offering an ideal setting to study the prognostic and predictive implications of these cells in many tumor types (6). Therefore, riboflavin accumulation detected as AF in tumors is a very specific and accurate marker of CSCs, which facilitates their isolation and characterization.

To this end, we conducted a pilot study to identify AF CSCs in a wide variety of 145 tumors. Importantly, AF+ cells were more tumorigenic than AF– cells, as we have previously shown (6), and AF was reduced when tumor cells were incubated with FTC, altogether confirming that the AF cells are *bona fide* CSCs and the AF observed was ABCG2 dependent (6). Based on the above findings, we set out to test the prognostic role of AF CSCs in resected colorectal tumors, as this tumor type represented the majority of the tumors analyzed. Previous studies have attempted to correlate CSCs with colorectal cancer outcome. More than 2 decades ago, the CSC marker CD133 was shown to be expressed in significantly higher levels in samples from patients with colorectal cancer with lymphatic invasion—this latter marker associated with a worse outcome (43). In 2011, Merlos-Suárez and colleagues (25) showed that the EphB2 receptor and the LRG5 marker (both markers present in adult intestinal stem cells, which become gradually silenced as they differentiate) could identify colorectal CSCs and predict disease relapse. Indeed, LGR5 has been characterized as one of the best stem cell population markers in colorectal cancer (44). Lgr5 identifies CSCs in genetically engineered mouse models that mimic the progression of human colorectal cancer (45). In fact, selective Lgr5+ cell ablation *in vivo* halts primary tumor growth, although targeting Lgr5+ CSCs has not been related to tumor regression (46). Moreover, it has been demonstrated that in tumors in which Lgr5+ cells are targeted, proliferative Lgr5– cells, via plasticity and dedifferentiation, restore the Lgr5+ CSC pool, leading to reinitiation of tumor growth (46, 47). High LGR5 expression has also been significantly associated with poor overall survival (OS; HR, 1.87; 95% CI, 1.23–2.84; *P* = 0.003) and with worse disease-free survival (DFS; HR, 2.44; 95% CI, 1.49–3.98; *P* = 0.001) in the recently published colorectal cancer meta-analysis (48). However, the role of LGR5 in colorectal cancer relapse and metastasis has been challenged in the past decade. Using mouse models of murine and human colorectal cancer, Fumagalli and colleagues (49) showed that circulating colorectal cancer cells were primarily Lgr5– and that the Lgr5– cells were responsible for distant metastases; however, via plasticity, Lgr5– cells become Lgr5+ CSCs in a niche-independent manner, restoring the hierarchy of the original tumor in metastatic lesions. With regard to relapse, Cañellas-Socias and colleagues (50) recently identified a unique tumor cell population known as high-relapse cells (HRC) that are neither differentiated nor stem-like but are responsible for metastatic relapse after surgical resection of the primary tumor in a murine model. These HRCs express epithelial membrane protein 1 (EMP1), a cell-to-cell adhesion molecule that facilitates their migration and colonization, and while not Lgr5+, the authors show that HRCs reacquire an LGR5+ stem cell and proliferation program at a later phase, necessary for metastatic outgrowth (50). Interestingly, we did not observe an overlap between AF and LGR5+ cells in colorectal cancer tumors, suggesting that AF cells represent a distinct CSC population in colorectal cancer like EMP1+ cells. Further studies are needed to determine the exact role of AF colorectal cancer CSCs in tumor relapse (and perhaps in metastasis) as well as whether AF colorectal cancer CSCs coexpress other relevant markers like EMP1.

A study by Ininuma and colleagues showed in a multi-institutional retrospective study including stage II and III colorectal cancer tumors that the detection of carcinoembryonic antigen, cytokeratin, and CD133 mRNA in plasma represented a significant prognostic factor for both OS (HR, 3.84; 95% CI, 2.41–6.22; *P* < 0.001) and DFS (HR, 3.02; 95% CI, 1.83–5.00; *P* < 0.001). These markers determined patients with a higher risk for recurrence and were linked to a worse prognosis (51). Our study, in contrast to the study by Ininuma and colleagues, is a prospective study, and more importantly, we determined the percentage of CSCs at the time of surgery with no additional postoperative determinations needed as compared with other studies such as that by Tie and colleagues (52), in which the authors show that ctDNA detection after stage II colorectal cancer resection provided direct evidence of residual disease and identified patients for very high risk of recurrence. Thus, at the time of surgery, using a simple non–antibody-based flow cytometric approach, we can accurately predict a patient’s risk for relapse, in conjunction with the other histopathologic factors, which can be used by the clinician to make more informative decisions about suitable treatment(s) and follow-up.

In our study, patients with H-AF showed a shorter DFS, and this risk significantly increased in the subpopulation of patients with positive LNs. Although AF combined with EpCAM was markedly better at determining the probability of recurrence-free survival, an adjusted hazards regression analysis showed that AF alone was better at determining the likelihood of relapse. We do not discard combining AF with EpCAM, as H-EpCAM/AF provided strong correlations but many times, did not reach significance. This may be due to the sample size of our study. Future analyses with larger patient numbers may resolve whether EpCAM should be used in combination with AF.

Our study is not without its limitations. First, we could not perform an OS analysis as the follow-up time has only been 5 years. Second, as only a single piece of approximately 0.5 to 0.8 cm^3^ for each surgically resected tumor was analyzed, our approach does not capture the inherent intratumoral heterogeneity that exists in many tumor entities and our results may have also been impacted by tumors with a high desmoplastic reaction or fibrotic stroma, inadvertently reducing the percentage of AF-positive cells detected. For future analyses, analyzing more than one tumor section or a pool of multiple postsurgical tumor biopsies should improve our detection capacity while at the same time take into consideration intratumoral heterogeneity. Third, as we did not initially set out to study AF cells exclusively in colorectal cancer, we did not include well-defined colorectal CSC markers in our analyses, such as LGR5, EMP1 (50), and/or EphB2 (53). Thus, future studies using our AF-based approach in colorectal cancer might benefit by combining AF with the aforementioned markers, although in two colorectal cancer tumors, we did not observe AF and LGR5 colocalization. Lastly, while clinically feasible, the detection of circulating AF CSCs was not performed in blood samples during the follow-up period to determine the effect of chemotherapy on these cells. However, this is the first study to confirm a consistent association between the percentage of CSCs and relapse as a prognostic factor in patients with colorectal cancer with positive LNs. Thus, our approach represents an important breakthrough in the field of prognostic markers not only for its accuracy but also for the simplicity and cost-effective technique. Our method to identify and potentially isolate this chemoresistant and aggressive population may also help investigators to determine new targets to fully eliminate them. Taken together, our results acknowledge the value of AF CSCs as a prognostic marker in resected colorectal cancer and, more importantly, as a cofactor in LN-positive tumors, which might help discriminate patients with a higher probability to develop resistance to conventional chemotherapy and who might undergo a more intense follow-up. Therefore, future studies should be directed in this direction, with the goal to incorporate AF CSC detection into prognostic systems/useful nomograms that could help physicians decide whether more intensive adjuvant therapy and/or follow-up should be offered to these patients. Considering that results from the IDEA study conducted in France showed that a longer course of oxaliplatin therapy was superior to a shorter course (6 vs. 3 months PFS) in the T4 and/or N2 subgroups (54), tumors with a higher percentage of AF CSCs might also benefit from a more intensive adjuvant treatment and a closer follow-up to detect recurrences.

Herein, we show that AF CSCs can be detected in freshly resected colorectal tumors and a high percentage of AF CSCs together with the presence of positive LNs is a strong predictor of disease recurrence. Drug election and treatment duration after colorectal tumor resection are based on clinical and biological markers, however, many are either inaccurate or nonspecific. Therefore, determining the presence of AF CSCs after tumor resection may represent an important tool in the management of these patients.

## Supplementary Material

Supplementary Tables Supplementary Table S1 showing the percentage of tumors with detectable AF cells and Table S2 with clinical and demographic characteristics of the CRC patients
