# Supplementary material for: Autofluorescent Cancer Stem Cells: Potential Biomarker to Predict Recurrence in Resected Colorectal Tumors
Source: Cancer Res Commun. 2024 Oct 2;4(10):2575–88. doi: 10.1158/2767-9764.CRC-24-0188 (PMC11445700; doi:10.1158/2767-9764.CRC-24-0188)
Supplement: Supplementary Tables — Supplementary Table S1 showing the percentage of tumors with detectable AF cells and Table S2 with clinical and demographic characteristics of the CRC patients [file crc-24-0188_supplementary_tables_suppst1-st2.docx]

# **Autofluorescent Cancer Stem Cells: Potential Biomarker to Predict Recurrence in Resected Colorectal Tumors**

Sonia Alcalá^1,2,*^, Gonzalo Serralta San Martin^3,4*^, Marta Muñoz-Fernández de Legaria^5^, Juan Moreno-Rubio^6,7^, Silvia Salinas^5^, Juan Carlos López-Gil^1,2^, José Alberto Rojo López^8^, Javier Martínez Alegre^4,8^, David Abraham Cortes Bandy^6^, Francisco Zambrana^4,6,7^, Ana-María Jiménez-Gordo^4,6,7^, Enrique Casado^4,6,7^, Miriam López-Gómez^4,6,†^, Bruno Sainz, Jr.^1,2,9†^

**Supplementary Tables**

**Supplementary Table S1: Percentage of tumors with detectable AF cells**

| **Tumor type** | **Number** | **Positive/Total** | **Percentage** |
| --- | --- | --- | --- |
| colon | 75 | 75/75 | 100 |
| kidney | 16 | 16/16 | 100 |
| stomach | 11 | 11/11 | 100 |
| breast | 10 | 7/10 | 70 |
| rectum | 5 | 5/5 | 100 |
| liver | 4 | 4/4 | 100 |
| larynx | 3 | 1/3 | 33 |
| uterus | 3 | 3/3 | 100 |
| ovarian | 2 | 2/2 | 100 |
| testicle | 2 | 2/2 | 100 |
| liposarcoma | 1 | 0/1 | 0 |
| lymphoma | 1 | 1/1 | 100 |
| gastric(*) | 1 | 1/1 | 100 |
| neuro-endocrine breast | 1 | 1/1 | 100 |
| caecum | 1 | 1/1 | 100 |
| colon metastasis from liver | 1 | 1/1 | 100 |
| ganglional metastasis from colon | 1 | 1/1 | 100 |
| liver metastasis from colorectal | 4 | 4/4 | 100 |
| liver metastasis from rectum | 1 | 1/1 | 100 |
| liver metastasis from small bowel NET | 1 | 1/1 | 100 |
| liver metastasis from neuroendocrine | 1 | 1/1 | 100 |

(*) mesenteric mass with jejunum infiltration

| **Supplementary Table S2: Clinical and demographic characteristics in patients with resectable CRC** | | | | | | |
| --- | --- | --- | --- | --- | --- | --- |
|  | | **Total sample** | **No relapse** | | **Relapse** | ***p-value*** |
|  | | **n=75** | **n=56** | | **n=19** |  |
| Patient characteristics | |  |  | |  |  |
| Sex – n (%) | |  |  | |  |  |
| Female | | 28 (37) | 21 (38) | | 7 (37) | ns |
| Male | | 47 (63) | 35 (63) | | 12 (63) | ns |
| Age - median (SD) | | 71 (12) | 72 (13) | | 71 (10) | ns |
| Tumor type n (%) | |  |  | |  |  |
| Colon | |  |  | |  |  |
| Right | | 34 (47) | 27 (49) | | 7 (41) | ns |
| Left | | 38 (54) | 28 (51) | | 10 (63) | ns |
| Transversal | | 3 (4) | 1 (2) | | 2 (11) | ns |
| Tumor stage n (%) | |  |  | |  |  |
| Stage I | | 13 (18) | 12 (22) | | 1 (6) | ns |
| Stage II | | 27 (36) | 22 (39) | | 5 (26) | *ns* |
| Stage III and N1 | | 35 (49) | 22 (41) | | 13 (76) | *p=0.01* |
| T factor n (%) | |  |  | |  |  |
| T1 | | 4 (6) | 4 (7) | | 0 (0) | ns |
| T2 | | 17 (23) | 14 (25) | | 3 (16) | ns |
| T3 | | 47 (66) | 35 (65) | | 12 (71) | ns |
| T4 | | 7 (10) | 3 (6) | | 4 (24) | *p=0.03* |
| Tumor histology n (%) | |  |  | |  |  |
| High histological grade | | 5 (7) | 3 (5) | | 2 (12) | ns |
| Perivascular invasion | | 11 (15) | 5 (9) | | 6 (11) | ns |
| Perineural infiltration | | 3 (4) | 1 (2) | | 2 (12) | ns |
| Microsatellite instability | | 5 (8) | 4 (8) | | 1 (7) | ns |
| CSC levels percent positive (IQR) |  | | |  | |  |
| AF | | 1.62 (0.3 - 7) | 1.55 (0.3 - 6.6) | | 1.93 (0.4 - 8.6) | ns |
| CD 90 | | 2.17 (1 - 6) | 2.4 (1.4 - 5.7) | | 1.1 (0.6 - 4.1) | ns |
| EpCAM | | 58.7 (42 - 74) | 58.4 (43 - 74) | | 60 (35 - 81) | ns |
| EpCAM/AF | | 0.48 (0 - 2) | 0.4 (0.04 - 1.8) | | 0.64 (0.8 - 2.3) | ns |
